# Supplementary material for: Generalization of fear learning is shaped by inhibitory sensory processing in mice
Source: Nat Commun. 2026 Apr 3;17:4825. doi: 10.1038/s41467-026-71356-5 (PMC13223235; doi:10.1038/s41467-026-71356-5)
Supplement: Supplementary file 1 — Supplementary Information [file 41467_2026_71356_MOESM1_ESM.pdf]

Supplementary Results

Behavioral data was compared to four different *a priori* models as described elsewhere (*60*). Briefly, four models of the distribution of time spent across odor zones were defined verbally here and quantitatively below.

- **No Fear Model**, conceptualized as equal time spent in all areas (a.k.a. chance model)
- **Fear Without Generalization Model**, conceptualized as minimal time spent in the CS zone, 50% reduced time in the CS-adjacent sides, and the balance of time distributed uniformly across all other regions
- **Fear Overgeneralization Model**, conceptualized as minimal time spent in all scented corners and the sides between them, 50% reduced time in the sides between an odor and the No Odor corner, and the balance of time distributed uniformly across all other regions
- **Gradient fear Model**, conceptualized as minimal time spent in the CS-zone, reduced time in the other scented corners in proportion to the similarity to the CS, reduced time in the sides between the CS odor and the other odors that is average of the dwell time in each corner, and the balance of time distributed uniformly across other regions.

The constraints on the models were that a) the minimum time spent in any corner was 5%, as needed to enter and identify the odorant, b) the sides of the arena were deemed to be the average of the time in the adjacent corners, and c) the similarity of MV to BA was treated as 66.67% and the similarity of MV to Hex was treated as 33.33%. All models had the same number of variables and only one parameter (a scaling parameter).

<u>*A priori* models:</u>

	No Fear	MV Fear Without Generalization	Overgeneralization	Proportional Fear
**Hex**	12.5%	15.83%	5%	15.4%
**MV-HEX**	12.5%	7.917%	5%	8.3%
**MV**	12.5%	5.00%	5%	5%
**MV-BA**	12.5%	7.917%	5%	6.3%
**BA**	12.5%	15.83%	5%	7.6%
**No-BA**	12.5%	15.83%	18.75%	15.3%
**No Odor**	12.5%	15.83%	37.5%	23.0%
**No-Hex**	12.5%	15.83%	18.75%	19.2%
Total	100	100	100	100

Based for each behavioral group, the average time spent across individuals (weighted by the inverse of their variance) was quantitatively compared to each of the four models, resulting in a residual sum of squares (RSS) and Akaike Information Criterion (AIC) score (*61*) indicating the goodness of fit. For each dataset, the relative likelihood of the best fitting model compared to each of the other models was computed from the AIC and presented below.

<u>Fear Conditioned Group, Vehicle control infusion</u>

Model (Fear Conditioned Group-VEHICLE)	RSS	AIC
Proportional Fear	21.3	14.24
Overgeneralization	35.2	18.24
CS-specific fear	39.3	19.13
No Fear	49.6	20.99

Comparison (Fear Conditioned Group-VEHICLE)	Likelihood Ratio (fold likelihood)
Proportional Fear vs Overgeneralization	7.39
Proportional Fear vs CS-Specific Fear	11.49
Proportional Fear vs No Fear	29.14

Fear Conditioned Group, $GABA_B$ blockade via CGP35348 infusion

Model (Fear Conditioned Group-CGP)	RSS	AIC
Overgeneralization	15.5	11.68
Proportional Fear	83.4	25.15
No Fear	101.0	26.69
CS-specific fear	107.9	27.21

Comparison (Fear Conditioned Group-CGP)	Likelihood Ratio (fold likelihood)
Overgeneralization vs. Proportional Fear	843.7
Overgeneralization vs. No Fear	1817.7
Overgeneralization vs. CS-Specific Fear	2359.9

Fear Conditioned Group, $GABA_B$ stimulation via ±baclofen infusion

Model (Fear Conditioned Group-Baclofen)	RSS	AIC
Proportional Fear	82.3	25.04
Overgeneralization	95.5	26.24
CS-Specific fear	56.5	22.0
No Fear	56.5	22.0

Comparison (Fear Conditioned Group-Baclofen)	Likelihood Ratio (fold likelihood)
No Fear vs CS-Specific Fear	1.004
No Fear vs Proportional Fear	4.51
No Fear vs. Overgeneralization	8.19

Non-conditioned Control Group, Vehicle control infusion

Model (Non-Conditioned Group-VEH)	RSS	AIC
Proportional Fear	82.0	25.02
Overgeneralization	124.1	28.34
CS-specific fear	42.4	19.74
No Fear	18.3	13.03

Comparison (Non-Conditioned Group-VEHICLE)	Likelihood Ratio (fold likelihood)
No Fear vs. CS-Specific Fear	28.7
No Fear vs. Proportional Fear	401.6
No Fear vs Overgeneralization	2110.5

Non-conditioned Control Group, $GABA_B$ blockade via CGP35348 infusion

Model (Non-Conditioned Group-CGP)	RSS	AIC
Proportional Fear	57.0	22.12
Overgeneralization	105.4	27.03
CS-specific fear	39.2	19.11
No Fear	3.2	-0.940

Comparison (Non-Conditioned Group-CGP)	Likelihood Ratio (fold likelihood)
No Fear vs. CS-Specific Fear	22,604
No Fear vs. Proportional Fear	101,505
No Fear vs Overgeneralization	1,183,600

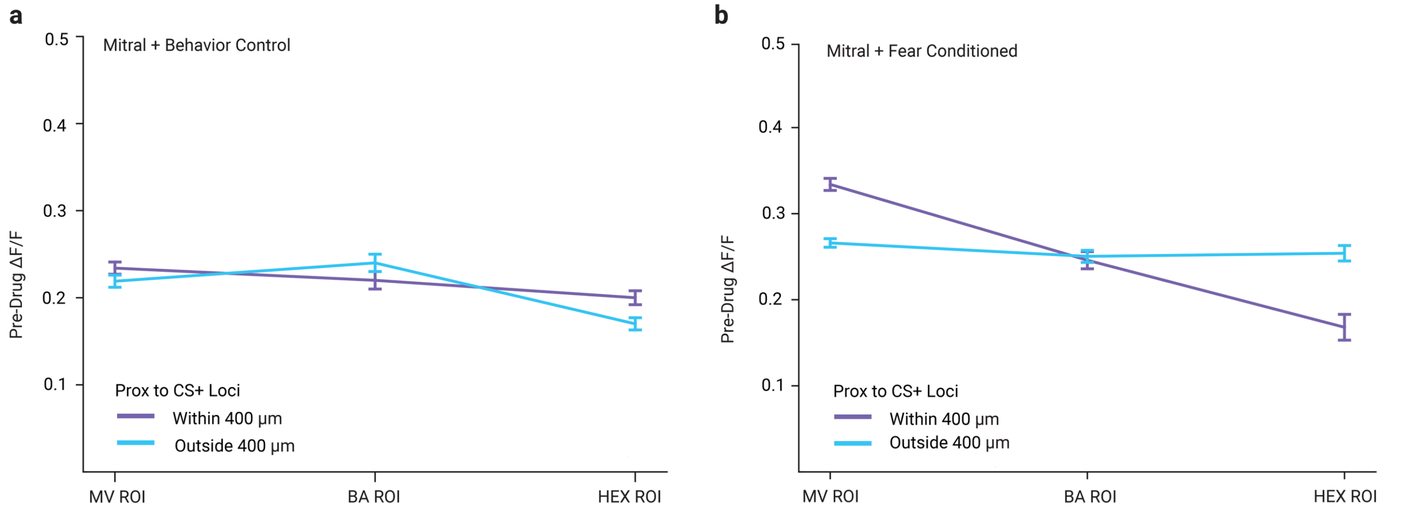

Fig. S1. Line graphs showing baseline (pre-drug) ΔF/F values for each odor in control (a) and paired (b) animals exhibiting fluorescence in mitral cells. Data is divided into two to show values for ROIs within (purple) or outside (blue) 400 µm (approx. diameter of 4 normal sized glomerulus) radius of the maximally responding MV ROI. In mice in the behavioral control groups (within 400 µm n = 275 ROI; outside 400 µm n = 482 ROI), there was no difference in the size of the odor-evoked responses across odors or across distances. However, in fear conditioned mice the size of the (pre-drug) odor responses depended on the distance, where MV ROIs within 400 µm (within n = 110 ROI; outside n = 139 ROI) of the MV response maximum showed a higher ΔF/F value, while closer HEX ROI (within n = 63 ROI; outside n = 184 ROI) showed lower ΔF/F (Fig. S1b; two-way ANOVA, interaction $F_{2,718} = 41.34$, $p < 0.001$, $\eta^2 = 0.089$). Data are presented as mean ± SEM. Source data are provided as a source data file.

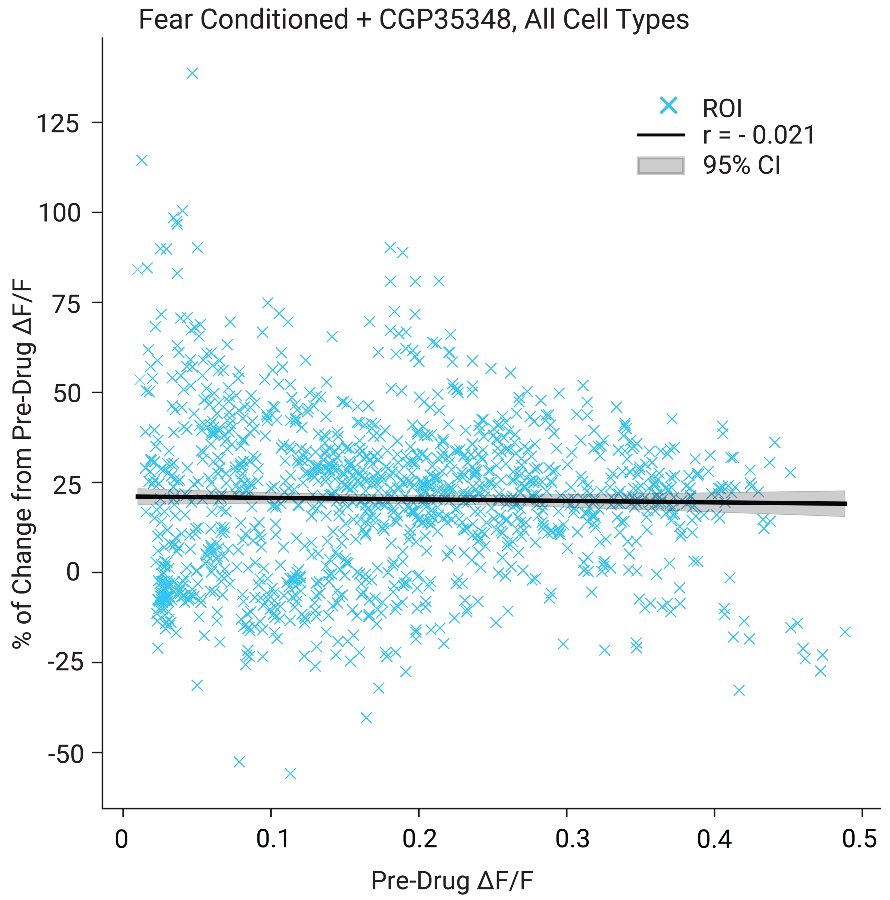

Fig. S2. Scatterplot showing relationship between pre-drug ΔF/F and percentage of increase after CGP35348 application in fear conditioned animals. Data points (blue x) show ROIs. Black line indicates the regression line (Pearson's r = -0.021, p = 0.47) with 95% confidence interval (gray highlight). The lack of correlation shows that the fluorescence increase difference we observed in different odors were not due to a ceiling effect. n = 1042 ROI from 14 mice. Source data are provided as a source data file.
