# Supplementary material for: Generalization of fear learning is shaped by inhibitory sensory processing in mice
Source: Nat Commun. 2026 Apr 3;17:4825. doi: 10.1038/s41467-026-71356-5 (PMC13223235; doi:10.1038/s41467-026-71356-5)
Supplement: Supplementary file 2 — Reporting Summary [file 41467_2026_71356_MOESM2_ESM.pdf]

nature portfolio

Corresponding author(s): Dr. Alper K. Bakir and Dr. John P. McGann

Last updated by author(s): 2-28-2026

Reporting Summary

Nature Portfolio wishes to improve the reproducibility of the work that we publish. This form provides structure for consistency and transparency in reporting. For further information on Nature Portfolio policies, see our Editorial Policies and the Editorial Policy Checklist.

Statistics

For all statistical analyses, confirm that the following items are present in the figure legend, table legend, main text, or Methods section.

n/a	Confirmed	
☐	☒	The exact sample size (*n*) for each experimental group/condition, given as a discrete number and unit of measurement
☐	☒	A statement on whether measurements were taken from distinct samples or whether the same sample was measured repeatedly
☐	☒	The statistical test(s) used AND whether they are one- or two-sided<br>*Only common tests should be described solely by name; describe more complex techniques in the Methods section.*
☐	☒	A description of all covariates tested
☐	☒	A description of any assumptions or corrections, such as tests of normality and adjustment for multiple comparisons
☐	☒	A full description of the statistical parameters including central tendency (e.g. means) or other basic estimates (e.g. regression coefficient) AND variation (e.g. standard deviation) or associated estimates of uncertainty (e.g. confidence intervals)
☐	☒	For null hypothesis testing, the test statistic (e.g. *F*, *t*, *r*) with confidence intervals, effect sizes, degrees of freedom and *P* value noted<br>*Give P values as exact values whenever suitable.*
☒	☐	For Bayesian analysis, information on the choice of priors and Markov chain Monte Carlo settings
☐	☒	For hierarchical and complex designs, identification of the appropriate level for tests and full reporting of outcomes
☐	☒	Estimates of effect sizes (e.g. Cohen's *d*, Pearson's *r*), indicating how they were calculated

Our web collection on statistics for biologists contains articles on many of the points above.

Software and code

Policy information about availability of computer code

Data collection	Imaging data were collected using Neuroplex by RedShirtImaging.
Data analysis	Data analysis was performed using custom scripts written in RStudio (v2024.02.0) and MATLAB (2019a), along with standard statistical functions. The analysis code is available at Zenodo (https://doi.org/10.5281/zenodo.18809357). The behavioral data was analyzed using EthoVision (XT 14)

For manuscripts utilizing custom algorithms or software that are central to the research but not yet described in published literature, software must be made available to editors and reviewers. We strongly encourage code deposition in a community repository (e.g. GitHub). See the Nature Portfolio guidelines for submitting code & software for further information.

Data

Policy information about availability of data

All manuscripts must include a data availability statement. This statement should provide the following information, where applicable:

- Accession codes, unique identifiers, or web links for publicly available datasets
- A description of any restrictions on data availability
- For clinical datasets or third party data, please ensure that the statement adheres to our policy

Source data underlying the figures are provided with this paper. Processed datasets and analysis code are available at Zenodo (https://doi.org/10.5281/zenodo.18809357). Raw imaging data are not publicly available due to their large file size.

Research involving human participants, their data, or biological material

Policy information about studies with human participants or human data. See also policy information about sex, gender (identity/presentation), and sexual orientation and race, ethnicity and racism.

Reporting on sex and gender	n/a
Reporting on race, ethnicity, or other socially relevant groupings	n/a
Population characteristics	n/a
Recruitment	n/a
Ethics oversight	n/a

Note that full information on the approval of the study protocol must also be provided in the manuscript.

Field-specific reporting

Please select the one below that is the best fit for your research. If you are not sure, read the appropriate sections before making your selection.

[x] Life sciences [ ] Behavioural & social sciences [ ] Ecological, evolutionary & environmental sciences

For a reference copy of the document with all sections, see nature.com/documents/nr-reporting-summary-flat.pdf

Life sciences study design

All studies must disclose on these points even when the disclosure is negative.

Sample size	Sample sizes were chosen based on prior experience with similar behavioral and imaging experiments and were sufficient to detect biologically meaningful effects.
Data exclusions	No collected data was excluded except for a very small number of trials with unambiguous optical artifacts. Because this was a learning study, mice that did not learn in the behavioral paradigm were excluded prior to data collection as described in the methods.
Replication	Each experimental group included multiple animals, with statistical comparisons between groups. The imaging data produced consistent outcomes across three mouse lines in which we visualized three different cell types. There is qualitative concordance between the data collected from the freezing and open field assays.
Randomization	Mice were randomly assigned to experimental groups.In the imaging data, the order of the odors were counterbalanced across subjects. In the accompanying behavioral data the order of the odors were randomized. In the open field experiment the locations of odors were counterbalanced across subjects.
Blinding	During the optical neurophysiology experiments and behavioral experiments, the experimenter was blind to the infusate (drug vs. vehicle). During the data analyses, the experimenters was blind to all experimental conditions.

Reporting for specific materials, systems and methods

We require information from authors about some types of materials, experimental systems and methods used in many studies. Here, indicate whether each material, system or method listed is relevant to your study. If you are not sure if a list item applies to your research, read the appropriate section before selecting a response.

Materials & experimental systems

n/a	Involved in the study	
[x]	[ ]	Antibodies
[x]	[ ]	Eukaryotic cell lines
[x]	[ ]	Palaeontology and archaeology
[ ]	[x]	Animals and other organisms
[x]	[ ]	Clinical data
[x]	[ ]	Dual use research of concern
[x]	[ ]	Plants

Methods

n/a	Involved in the study	
[x]	[ ]	ChIP-seq
[x]	[ ]	Flow cytometry
[x]	[ ]	MRI-based neuroimaging

Animals and other research organisms

Policy information about studies involving animals; ARRIVE guidelines recommended for reporting animal research, and Sex and Gender in Research

Laboratory animals	Adult mice (C57BL/6J background; 68 male and 53 female; 3–9 months of age) were used in this study. Transgenic mouse lines with Cre (omp, gad2, tbet) recombinase–mediated conditional expression of GCaMP6f were used for imaging experiments (bred in-house), and additional wild-type C57BL/6J mice were used for non-imaging experiments.
Wild animals	n/a
Reporting on sex	Both male and female mice were used. Sex was not a primary factor in the original study design but was evaluated in statistical analyses where appropriate. No significant sex differences were observed in behavioral or neurophysiological measures. Sex-disaggregated data for behavioral experiments are shown in Fig. 1c. For the open-field test, sample sizes were insufficient to support additional sex-disaggregated analyses in accordance with journal guidelines.
Field-collected samples	n/a
Ethics oversight	The work was performed in compliance with protocols approved by the Rutgers University Institutional Animal Care and Use Committee (IACUC, protocol #09-022).

Note that full information on the approval of the study protocol must also be provided in the manuscript.

Plants

Seed stocks	n/a
Novel plant genotypes	n/a
Authentication	n/a
